# Childhood leukaemia and ordnance factories in west Cumbria during the Second World War

**DOI:** 10.1038/sj.bjc.6603199

**Published:** 2006-06-06

**Authors:** L Kinlen

**Affiliations:** 1 Cancer Research UK Epidemiology Unit, University of Oxford, Richard Doll Building, Roosevelt Drive, Headington, Oxford OX3 7LF, UK

**Keywords:** leukaemia, childhood, epidemiology, population mixing, infection, construction, rural, wartime

## Abstract

Much evidence has accumulated that childhood leukaemia (CL) is a rare response to a common, but unidentified, infection and in particular that situations involving the unusual mixing of urban and rural groups (approximating to, respectively, groups infected with, and susceptible to, the relevant microorganism) can produce localised epidemics with consequent increases of the infrequent leukaemic complication. During the Second World War, explosives production factories were built and operated at Drigg and Sellafield, and a shell filling factory at Bootle, in west Cumbria, England, requiring substantial numbers of construction workers to be brought into this remote and isolated area. Following the design of an earlier study of CL near large (post-war) rural construction sites, mortality from this disease was investigated with the help of the Office of National Statistics, in the area around these Cumbrian factories where local workers largely lived, during the construction period and with particular reference to the overlapping construction and operational phase when the mixing of local and migrant workers would have been greatest. An excess of leukaemia deaths at ages 1–14 was found during the construction period (observed 3; observed/expected (O/E) 2.2, 95% confidence interval (CI): 0.6, 6.0), which was more marked and statistically significant during the overlap with operations (O 3; O/E 4.5, 95% CI: 1.1, 12.2), especially at ages 1–4 (O 2; O/E 7.1, CI: 1.2, 23.6). A previous investigation did not detect this excess because it considered only a small part of west Cumbria that omitted the communities where most of the workforce lived, having incorrectly attributed the post-war expansion of the village of Seascale (situated between Drigg and Sellafield) to the wartime ordnance factories. The present findings are consistent with the results of the earlier study of rural construction projects and with the general evidence that marked rural–urban population mixing increases the risk of CL.

The well known excesses of childhood leukaemia (CL) in Seascale and Thurso near the nuclear sites at, respectively, Sellafield in Cumbria, NW England and Dounreay in Caithness, northern Scotland both involved remote and isolated areas that had experienced major population movements (Kinlen, 1988, 1995, 2000). Such unusual mixing of urban and rural populations would promote what is, in fact, central for an infective epidemic, namely increased contacts between infected and susceptible individuals (the latter being more prevalent in rural areas) and it led to the (infective) population mixing (PM) hypothesis of CL. This holds that in contrast to the usual pattern of (sporadic) infection, marked rural–urban PM produces a localised epidemic of the infection of which CL is a rare manifestation, with consequent excess cases of its uncommon leukaemic complication, particularly below age five. The evidence overall supports CL being a rare response to a common, but unidentified, infection and that it is increased by marked rural–urban PM (Kinlen, 1995, 2000; Doll, 1999; Dickinson and Parker, 1999), as in the post-war creation of rural new towns (Kinlen, 1988; Kinlen *et al*, 1990) and the wartime evacuation of urban children to the countryside (Kinlen and John, 1994). The important role of adults in transmitting this (mainly subclinical) infection has been highlighted in rural PM studies involving large numbers of military servicemen (Kinlen and Hudson, 1991; Kinlen and Balkwill, 2001; Kinlen and Doll, 2004), construction and oil workers (Kinlen *et al*, 1993, 1995).

The Committee on Medical Aspects of Radiation in the Environment (COMARE) in its Fourth Report (1996), on the basis of a study that it had commissioned and has since been published (Cartwright *et al*, 2001), cast doubt upon PM as a cause of CL (and, in particular, as the cause of the Seascale excess after 1950). This was in large part because no case of CL had been found in Seascale during the Second World War, when two ordnance factories were built and operated at Sellafield and nearby Drigg, which, COMARE and Cartwright *et al* (2001) incorrectly believed, had involved a large influx of people into Seascale. The negative influence of COMARE's views upon opinion on the PM hypothesis, together with the limitations of its study, has prompted a detailed examination of the evidence.

## MATERIALS AND METHODS

### The Royal Ordnance Factories (ROFs)

**Figure 1:**
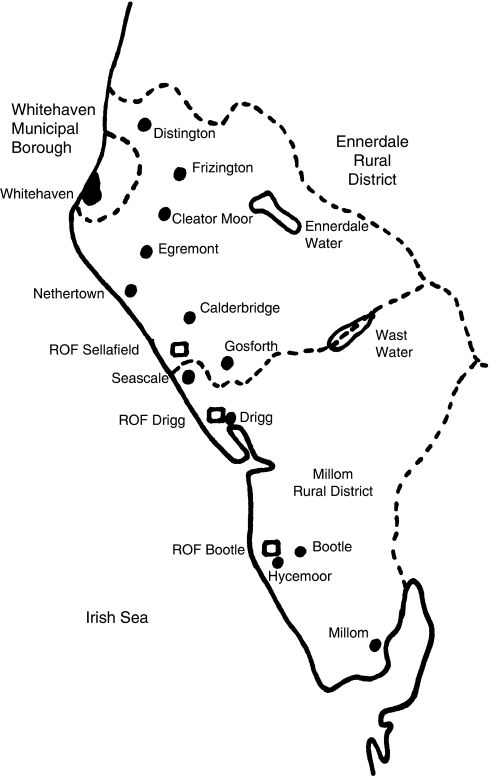
Map of west Cumbria, England, showing the locations of the ROFs at Drigg, Sellafield and Bootle, and neighbouring communities, in relation to the three local authority areas Ennerdale RD, Millom RD and Whitehaven MB.

The explosives ROFs in Cumbria were sited at Drigg and Sellafield, about 6 km apart, and manufactured trinitrotoluene (TNT). These isolated and remote coastal sites were chosen because of the hazardous nature of the process and to minimise the risk of enemy air attack. Construction of ROF Drigg (in the north of Millom Rural District (RD), see Figure 1) began in early 1940, with TNT produced from March 1941 when the factory was far from complete; an appreciable amount of civil engineering work was carried out in parallel with production because of the great need for TNT. Construction of ROF Drigg was essentially complete at the end of 1941 when it achieved its target production of 400 ton per week (Smith, 1946). Construction of ROF Sellafield (in the south of Ennerdale RD, see Figure 1) began later in early 1942 with TNT production starting in February 1943. The Superintendent of ROF Drigg was responsible for both sites. During 1940, about 4000 construction workers were employed at ROF Drigg and a similar number later at ROF Sellafield. When complete, the two ROFs employed almost 3000 workers, with much of the production workforce recruited initially from the local construction workforce; from mid-1942, women played an increasingly large role. TNT production ceased in August 1945. (Except where otherwise indicated, the above details are taken from the National Archives document on the history of ROF Drigg (Anon, 1946).)

In 1941–42, a third munitions ROF, overlooked by COMARE (1996), was built at Hycemoor, Bootle in Millom RD on the coastal railway line some 11 km south of Drigg and next to the pre-existing Eskmeals Proof Range, initially as a shell filling factory (Anon, 1941, 1942a) before becoming a shell breaking-down facility in 1944 (Anon, 1946). Filling operations had started by August 1942 and required a workforce of around 450 (Anon, 1942a). As a breaking-down factory, ROF Bootle also came under the Superintendent of ROF Drigg (Anon, 1946).

### Other population mixing

Other wartime activities would have further contributed to PM in the area, including the construction and operation of RAF Millom and the aircraft components factory of High Duty Alloys Ltd at Distington (in Ennerdale RD), as both involved the import of people from outside west Cumbria. The area also received children evacuated from towns and cities (see Discussion).

### Study areas

Unskilled labour for the construction of the ROFs was recruited locally, mainly from Whitehaven, Cleator Moor (in Ennerdale RD) and the neighbouring communities that had been severely affected by unemployment during the inter-war industrial depression (Anon, 1946). These men came by train to the stations at Sellafield and Drigg and by special buses to the factory gates. Skilled men, and also additional construction workers when building and operations were concurrent, were required from outside the area. Local hostel accommodation was provided, initially through the requisition in 1940 of the youth hostel (Stanley Ghyll House) in Eskdale, and then by the erection of a purpose-built hostel at Greengarth Hall, Holmrook (adjacent to Drigg) for up to 500 men, which was completed in Spring 1941, meeting the accommodation requirements of parallel construction and production work at ROF Drigg (Anon, 1946). Greengarth Hall hostel continued to function throughout the war, but below capacity after the construction of ROF Drigg was completed at the end of 1941. Workers, mainly those involved in production, were also billeted locally, while a few score police guards were housed in huts just outside the main gate of ROF Drigg. The number of professionals (mainly chemists and engineers) was relatively small and many lived at Irton Hall, Holmrook (with a capacity of about 50) or in the Sellafield Staff Club, an annex to the Scawfell Hotel at Seascale (with a capacity of around 40) (Anon, 1946). Some bungalows were built for married workers: 12 at Drigg, 24 at Seascale and 17 at Yottenfews (situated to the north of Sellafield towards Calderbridge) (Anon, 1945, 1946, 1948, 1949).

During the period of peak labour demand in 1941–42, when construction and production were concurrent, up to 500 building workers from outside the area (many from Ireland) were accommodated at a temporary camp at Nethertown (about 3 km from Egremont), next to the Nethertown railway station about 6 km north of Sellafield, for the construction of ROF Sellafield (Anon, 1942b). This camp was abandoned when this ROF began operations early in 1943 (Anon, 1942b) although it was re-used after the war in the building of nuclear facilities (Anon, 1953). Construction workforce details for ROF Bootle during 1941–42 are not readily available, but it may be presumed that workers from outside west Cumbria were required to supplement local labour as construction was also underway at ROFs Drigg and Sellafield. Near ROF Bootle, Wellbank Hall hostel, for up to 500 (presumably mainly construction) workers, was erected between Hycemoor and Bootle, and the 1945 electoral register shows about 50 houses in Bootle additional to those in the 1939 register.

As the workers involved in building and operating the Cumbrian ROFs lived over a wide area, the study area was defined as Whitehaven Municipal Borough (MB), Ennerdale RD and Millom RD, the only (former) local authority areas to be greatly affected. These three areas constituted southwestern Cumberland, and currently comprise Copeland County District in the present-day county of Cumbria.

### Study periods and age-specific populations

The study periods were defined as in an earlier study of large post-war rural construction projects (Kinlen *et al*, 1995) as follows: (a) the construction period together with the calendar year immediately following, that is, 1940–43, henceforth termed the ‘construction period’; (b) the period of greater PM in which construction overlapped with the operation of the ROFs, that is, 1941–42. As in that earlier study, children under the age of 15 years were covered with particular focus on those below age of five when the leukaemic effect of PM is usually greatest, their fewer opportunities for previously meeting the relevant infection leaving more of them susceptible (Kinlen, 1995).

The closest date to the study period for which age-specific populations by local authority area are available is September 1939, when national registration was carried out at the outbreak of war (National Register, 1950). As stressed in this publication, local populations had already been affected by the appreciable evacuation of urban centres that had just taken place.

The study populations were derived by two methods: in the first (Method A), as the official evacuation scheme involved much larger numbers of children aged 5–14 than 0–4 years, the children aged 0–4 registered in the three areas were (conservatively) regarded as all being ‘local’, even though they would have included some evacuees. The local populations in the 0–4-year age group in each of the years 1940–43 were derived by linear interpolation using the 1939 and 1947 national registration population estimates (General Register Office, 1949; National Register, 1950). In Method B, live births in the three areas in the years 1936–43 were regarded as all being to local families and the numbers of children aged 0–4 in the years 1940–43 (less neonatal deaths) were approximated using a birth cohort approach and applying annual national mortality rates by single years of age.

Owing to the effect of evacuation upon the registered numbers of older children, the numbers of local children aged 5–14 in the study area were derived from the two sets of population estimates for those aged 0–4, using the ratios of the national numbers at ages 0–4 to those aged 5–9 and 10–14. The populations of children aged 1–4 (see below) in the three districts were taken as the same proportion of the 0–4 age group as in the country as a whole (around four-fifths).

### Observed and expected numbers of deaths

After special consideration, the Office for National Statistics (ONS) agreed to identify CL deaths (although withholding names) in Millom and Ennerdale RDs and in Whitehaven MB in the period 1940–43. The death registers for the Millom and Whitehaven registration districts (covering the study area) were searched by ONS staff for any entries at ages 1–14 years with a mention of leukaemia or aleukaemia. It was at the request of ONS that infant deaths (below age 1 year) were not covered, because of the difficulty of scanning the often poorly legible details on microfilm of the many infant deaths. In any case, this omission is not serious as there were only 67 infant leukaemia deaths in the whole country in the study period, so that less than 0.2 was expected in the study. To determine whether any CL deaths from the study area had been registered near the county's only sizeable hospital (in Carlisle, outside the study area), the death registers in that city were also checked.

Expected (E) numbers of deaths in each of the years 1940–43 were derived by multiplying the above two sets of age-specific population estimates by the corresponding mortality rates for leukaemia in England and Wales. Observed (O) and expected (E) numbers were compared and exact Poisson 95% confidence intervals (CI) calculated for O/E ratios. As it is known that conventional ‘exact’ Poisson CI are overly conservative for small observed numbers, exact Poisson CI were calculated using the Mid-P method (Cohen and Yang, 1994; Kulkarni *et al*, 1998).

## RESULTS

As shown in Table 1, three deaths from CL at ages 1–14 years were recorded among residents of the study area during the construction period (1940–43). All three deaths occurred in local children; no case was found in an evacuee (or in the Carlisle registers). When using Method A to calculate local populations of children, the observed number of 3 compares with 1.37 expected, an excess that is not statistically significant. However, all three deaths occurred in the overlapping construction-operational period (1941–42) during which the excess was statistically significant (*P*<0.05) both overall at ages 1–14 years (O 3, E 0.67; O/E 4.5, 95% CI: 1.1, 12.2) and in those aged 1–4 years (O 2, E 0.28; O/E 7.1, 95% CI: 1.2, 23.6), as shown in Table 2. The results obtained using Method B are shown in footnotes to the tables (e.g. during 1941–42: at ages 1–14 years, O/E 5.2, 95% CI: 1.3, 14.1; and at 1–4 years, O/E=8.0, 95% CI: 1.3, 26.4). Although the O/E ratios obtained by Method B are somewhat greater than those by Method A, the associated levels of statistical significance are similar.

## DISCUSSION

The increase in CL mortality found here in relation to the wartime ROFs in Cumbria is consistent with the PM hypothesis (see Introduction), and also with the findings of an earlier study of large rural construction projects in the period 1945–94 (Kinlen *et al*, 1995). Indeed, the ROFs met the criteria used in that study, but the series of CL death certificates, on which it depended, only began in 1945. In both, the study periods were defined similarly: the construction period plus the succeeding calendar year, and the period of concurrent construction and operations when PM was predicted to be maximal. In that investigation, study areas were defined in two ways: the area within 10 km of the construction project and, in the case of a (Scottish) county with multiple hydroelectric schemes, the whole of that county so as to embrace the home areas of local workers (Kinlen *et al*, 1995). Applying the first definition to the Cumbrian ROFs would produce a low-powered study through failing to include Whitehaven, Cleator Moor and neighbouring communities, singled out for contemporary mention as places where many workers lived (Anon, 1946). The present approach of focusing on the main areas of residence of the local workers was also used in PM study of the North Sea oil industry (Kinlen *et al*, 1993).

West Cumbria, situated on the extreme northwest coast of England beyond the Lake District, was notably remote and isolated before the Second World War. The barrier to communications presented by the Cumbrian Mountains helped to make its people, including those in the recession-hit town of Whitehaven, effectively a separate and self-contained population with little contact with those living beyond these mountains. The two RDs and the town of Whitehaven have been considered as an entity, since by sharing in the isolation and remoteness of this coastal strip, all would tend to have a higher than average proportion of susceptible residents. In the same way, some isolated towns (like Thurso and Lerwick) were included within the rural study areas affected by the oil industry and large construction projects (Kinlen *et al*, 1993, 1995). The rapid construction of the wartime ROFs in the area would have produced unaccustomed PM, and the associated CL excess in the part from which local workers were mainly drawn points to its cause in this PM. This is especially so given the statistically significant excess of CL in the years 1941–42 when construction and operations overlapped and PM would have been maximal. In the earlier study of large rural construction projects, the excess risk in this overlap period was also greater than in the construction period as a whole (Kinlen *et al*, 1995).

The wartime evacuation of children from population centres to safer places was associated with an increase of CL in rural reception areas (Kinlen and John, 1994). It might be argued, therefore, that at least some of the increase observed in the present study was attributable to evacuees. Three considerations weigh against this: first, the excess of CL associated with evacuation involved only local children aged 5–14 years (the age group of most evacuated children) whereas the present excess (especially in the overlapping construction-operational period) mainly affected younger children. Second, the excess involved both the town of Whitehaven and the adjoining RDs, being significant in the former (see Table 2), whereas no increase of CL was found in urban areas as a whole that received many evacuees. More importantly, all three CL cases occurred in the two local authority areas that, in the evacuation study, belonged to the ‘low’ evacuee category, which showed no excess.

It is not surprising that no effect of the ROFs on CL risk in Seascale could be detected in the study of COMARE (1996) and Cartwright *et al* (2001). The village was relatively unaffected by the ROFs, as the 1939 and 1945 electoral registers confirm, with only 24 bungalows built during the war, other accommodation for temporary workers being limited and not for families. Any children resident in the bungalows erected in the war years at Drigg (12) and Yottenfews (17) would have attended the nearby village schools in Drigg and Calderbridge rather than Seascale school. Workers from outside the area were mainly accommodated away from Seascale at hostels near Drigg and Bootle, or Nethertown construction camp to the north of Sellafield and would have mixed at work with local workers who were drawn mainly from communities north of the ROFs. This contrasts with the marked impact on Seascale of the Windscale nuclear facility built at Sellafield after the war. Electoral registers confirm that the population increase in Seascale only began in 1950 when many workers’ families started moving into the new purpose-built houses, leading to a 10-fold increase in primary school numbers there during the 1950s and 1960s. But during the war, Seascale was a small village; in each of the years 1941–44, its school roll showed no more than 41 children up to age 15 (Kinlen *et al*, 1997). As a consequence, the expected number of CL cases in Seascale during the war was less than 0.02 and a single death would have represented a 50-fold excess. Statistical power was not improved by COMARE (1996) and Cartwright *et al* (2001) including Gosforth, this parish also being little affected by the ROFs.

The view of Cartwright *et al* (2001) and of COMARE in its Fourth Report (1996) that the absence of a case of CL in Seascale during the war provides evidence against the PM hypothesis was evidently influenced by their incorrectly attributing details of the extensive post-war housing construction in Seascale to the war years. Thus, COMARE (1996, p 114, 116) reported the building of ‘196 houses in Windscale/Sellafield’ between 1941 and 1946 in the belief that ‘Sellafield/Windscale … [was] part of Seascale parish’, whereas it was 3 km to the north of Seascale across the River Calder in the parish of St Bridget's, Beckermet (in Ennerdale RD), which is not even adjacent to Seascale parish. But a more important error was in relating extensive housing development to the war years when in fact it was post-war – the consequence of misreading the date of a National Archives document (Anon, 1949) as 1946 (COMARE, 1996 p. 114) although it was 1 August 1949. The figure of 196 houses consists of 12 bungalows at Drigg, four pre-war houses at Sellafield and Drigg, and 180 houses clearly marked in the document as ‘under construction’ in 1949. These 180 houses were, in fact, the first phase of the post-war expansion of Seascale to house the families of Windscale workers, as electoral registers confirm. Consequently, COMARE and Cartwright *et al* (2001) considered the absence of CL in Seascale during the war in the mistaken belief that the village received a large population influx in these years.

In conclusion, the excess of CL in west Cumbria associated with the ROFs at Drigg, Sellafield and Bootle during the Second World War, especially in their overlapping construction-production period, accords with an earlier study of large rural construction projects and with the broader evidence that marked rural–urban PM increases CL risk, particularly below age five. It is not surprising that the excess is apparent in the wider area where the local workers largely lived rather than in Seascale, for in contrast to the major effects on it of the post-war nuclear facility at Sellafield, this village was little affected by the factories.

## Figures and Tables

**Table 1:** Childhood leukaemia mortality in west Cumbria by age and local authority area, in relation to the construction of ordnance factories in 1940–43: observed (O) and expected (E) numbers of deaths, O/E ratios and exact Poisson 95% confidence intervals (CI)

	**Age group**								
	**1–4 years**			**5–14 years**			**Total (1–14 years)**		
**Local authority area**	**O**	**E** *	**O/E (95% CI)**	**O**	**E** *	**O/E (95% CI)**	**O**	**E** *	**O/E (95% CI)**
Ennerdale and Millom RDs	1	0.37	2.70 (0.14, 13.33)	0	0.49	0 (0.00, 6.11)	1	0.86	1.16 (0.06, 5.73)
Whitehaven MB	1	0.22	4.55 (0.23, 22.42)	1	0.29	3.45 (0.17, 17.01)	2	0.51	3.92 (0.66, 12.96)
West Cumbria (Total)	2	0.59	3.39 (0.57, 11.20)^a^	1	0.78	1.28 (0.06, 6.32)^b^	3	1.37	2.19 (0.56, 5.96)^c^

CI=confidence intervals; MB=Municipal Borough; RD=Rural District.

* Expected numbers of deaths, E, calculated by Method A (see Materials and Methods). By Method B, the O/E ratios and 95% CI are ^a^3.70 (0.62, 12.24), ^b^1.47 (0.07, 7.25); and ^c^2.46 (0.63, 6.69).

**Table 2:** Childhood leukaemia mortality in west Cumbria by age and local authority area, in relation to the overlapping construction and operational phase of ordnance factories during 1941–1942: observed (O) and expected (E) numbers of deaths, O/E ratios and exact Poisson 95% confidence intervals (CI)

	**Age group**								
	**1–4 years**			**5–14 years**			**Total (1–14 years)**		
**Local authority area**	**O**	**E** *	**O/E (95% CI)**	**O**	**E** *	**O/E (95% CI)**	**O**	**E** *	**O/E (95% CI)**
Ennerdale and Millom RDs	1	0.17	5.88 (0.29, 29.01)	0	0.24	0 (0.00, 12.48)	1	0.41	2.44 (0.12, 12.03)
Whitehaven MB	1	0.11	9.09 (0.45, 44.84)	1	0.15	6.67 (0.33, 32.88)	2	0.26	7.69 (1.29, 25.41)
West Cumbria (Total)	2	0.28	7.14 (1.20, 23.60)^a^	1	0.39	2.56 (0.13, 12.65)^b^	3	0.67	4.48 (1.14, 12.19)^c^

CI=confidence intervals; MB=Municipal Borough; RD=Rural District.

* Expected numbers of deaths, E, calculated by Method A (see Materials and Methods). By Method B, the O/E ratios and 95% CI are ^a^8.00 (1.34, 26.43), ^b^3.03 (0.15, 14.95) and ^c^5.17 (1.32, 14.08).

## References

[bib1] Anon (1941) Filling Factory Construction Programme. In: New Small Filling Factories Nos. 10/20. National Archives Catalogue Reference No. WO 185/18. Kew, UK: The National Archives

[bib2] Anon (1942a) Labour Requirements: Royal Ordnance Factories. National Archives Catalogue Reference No. AVIA 22/1161. Kew, UK: The National Archives

[bib3] Anon (1942b) Royal Ordnance Factory, Sellafield, Cumb. construction camp: proposed conversion to hostel for Royal Ordnance Factory workers. National Archives Catalogue Reference No. AVIA 22/1269. Kew, UK: The National Archives

[bib4] Anon (1945) R.O.F. Drigg/Sellafield/Bootle. Buildings and Rentals. In: Royal Ordnance Factory Sellafield, Cumb.: Disposal. National Archives Catalogue Reference No. AVIA 22/408. Kew, UK: The National Archives

[bib5] Anon (1946) The History of Royal Ordnance Factory Drigg National Archives Catalogue Reference No. SUPP 5/956. Kew, UK: The National Archives

[bib6] Anon (1948) Windscale: housing. National Archives Catalogue Reference No. AB 8/271. Kew, UK: The National Archives

[bib7] Anon (1949) List of married quarters, August 1st, 1949. In: Housing and Hostels National Archives Catalogue Reference No. AVIA 46/308. Kew, UK: The National Archives

[bib8] Anon (1953) Windscale: Housing and Canteen Facilities National Archives Catalogue Reference No. AB 16/784. Kew, UK: The National Archives

[bib9] Cartwright RA, Dovey GJ, Kane EV, Gilman EA (2001) The onset of the excess of childhood cancer in Seascale, Cumbria. J Publ Health Med 23: 314–322, correspondence (2002) 24: 342–344. 10.1093/pubmed/23.4.314 11873895

[bib10] Cohen GR, Yang S-Y (1994) Mid-P confidence intervals for the Poisson expectation. Stat Med 13: 2189–2203 7846419 10.1002/sim.4780132102

[bib11] Committee on Medical Aspects of Radiation in the Environment (COMARE) (1996) Fourth Report. The Incidence of cancer and leukaemia in young people in the vicinity of the Sellafield site, West Cumbria: further studies and an update of the situation since the publication of the report of the Black Advisory Group in 1984. Wetherby, UK: Department of Health

[bib12] Dickinson HO, Parker L (1999) Quantifying the effect of population mixing on childhood leukaemia risk: the Seascale Cluster. Br J Cancer 81: 144–151 10487626 10.1038/sj.bjc.6690664 PMC2374359

[bib13] Doll R (1999) The Seascale cluster: a probable explanation. Br J Cancer 81: 3–5 10487604 10.1038/sj.bjc.6690642 PMC2374279

[bib14] General Register Office (1949) Estimates of the sex and age distribution of the civilian population in regions and administrative areas of England and Wales at 31st December 1947. London: HMSO

[bib15] Kinlen L (1988) Evidence for an infective cause of childhood leukaemia: comparison of a Scottish New Town with nuclear reprocessing sites in Britain. Lancet ii: 1323–1327 10.1016/s0140-6736(88)90867-7 2904050

[bib16] Kinlen LJ (1995) Epidemiological evidence for an infective basis in childhood leukaemia. Br J Cancer 71: 1–5 7819022 10.1038/bjc.1995.1 PMC2033475

[bib17] Kinlen LJ (2000) Infection, childhood leukaemia and the Seascale cluster. Radiol Protect Bull 226: 9–18

[bib18] Kinlen LJ, Balkwill A (2001) Infective cause of childhood leukaemia and wartime population mixing in Orkney and Shetland, UK. Lancet 357: 858 11265959 10.1016/s0140-6736(00)04208-2

[bib19] Kinlen LJ, Clarke K, Hudson C (1990) Evidence from population mixing in British New Towns 1946–85 of an infective basis for childhood leukaemia. Lancet 336: 577–582 1975376 10.1016/0140-6736(90)93389-7

[bib20] Kinlen LJ, Craft AW, Parker L (1997) The excess of childhood leukaemia near Sellafield: commentary on the fourth COMARE report. J Radiol Protect 17: 63–71

[bib21] Kinlen LJ, Dickson M, Stiller CA (1995) Childhood leukaemia and non-Hodgkin's lymphoma near large rural construction sites, with a comparison with Sellafield nuclear site. BMJ 310: 763–768 7711579 10.1136/bmj.310.6982.763 PMC2549162

[bib22] Kinlen L, Doll R (2004) Population mixing and childhood leukaemia: Fallon and other US clusters. Br J Cancer 91: 1–3 15226760 10.1038/sj.bjc.6601982 PMC2364755

[bib23] Kinlen LJ, Hudson C (1991) Childhood leukaemia and poliomyelitis in relation to military encampments in England and Wales in the period of national military service, 1950–63. BMJ 303: 1357–1362 1760600 10.1136/bmj.303.6814.1357 PMC1671618

[bib24] Kinlen LJ, John SM (1994) Wartime evacuation and mortality from childhood leukaemia in England and Wales in 1945-9. BMJ 309: 1197–1202 7987150 10.1136/bmj.309.6963.1197 PMC2541692

[bib25] Kinlen LJ, O'Brien F, Clarke K, Balkwill A, Matthews F (1993) Rural population mixing and childhood leukaemia: effects of the North Sea oil industry in Scotland, including the area near Dounreay nuclear site. BMJ 306: 743–748 8490337 10.1136/bmj.306.6880.743 PMC1677226

[bib26] Kulkarni PM, Tripathi RC, Michalek JE (1998) Maximum (Max) and Mid-P confidence intervals and *P* values for the standardized mortality and incidence radios. Am J Epidemiol 147: 83–86 9440403 10.1093/oxfordjournals.aje.a009371

[bib27] National Register, United Kingdom and Isle of Man (1950) Statistics of Population on 29th September 1939. Report and Tables. London: HMSO

[bib28] Smith DM (1946) Construction of Explosive ROFs. National Archives Catalogue Reference No. CAB 102/628. Kew, UK: The National Archives

